# Revisiting Effective Communication Between Patients and Physicians: Cross-Sectional Questionnaire Study Comparing Text-Based Electronic Versus Face-to-Face Communication

**DOI:** 10.2196/16965

**Published:** 2020-05-13

**Authors:** Tala Mirzaei, Nicole Kashian

**Affiliations:** 1 Department of Information Systems and Business Analytics Florida International University Miami, FL United States; 2 Department of Communication Florida International University Miami, FL United States

**Keywords:** telemedicine, telemedicine, eHealth, text telecommunication, health communication

## Abstract

**Background:**

Research has shown that text-based communication via telemedicine will continue to be a mode of communication that patients and physicians use in the future. However, very few studies have examined patients’ perspectives regarding the increased use of text-based communication versus face-to-face (FtF) communication.

**Objective:**

This study aimed to understand and compare the potential differences in patients’ perceptions of communication effectiveness with their physicians through different modes of communication.

**Methods:**

We conducted a web-based survey of 345 patients to explore the impact of different channels on effective communication and perceived health behavior and outcomes. We tested the impact of patients’ perceived communication and media effectiveness on their self-efficacy, communication satisfaction, and perceived health outcomes, separately for text-based information technology (IT)–mediated communication and FtF communication. Furthermore, we conducted a group comparison to identify significant differences across these 2 groups.

**Results:**

We found no significant differences between patients’ perceptions of effective communication using either IT-mediated communication or FtF communication with their physicians. However, we found significant differences in patients’ perception of media effectiveness: patients perceived FtF communication to be a more favorable medium (*P*=.02). Interestingly, we found no significant difference in terms of benefits (*P*=.09) and success (*P*=.08) of IT-mediated communication versus FtF communication.

**Conclusions:**

The results of this study imply that patients can achieve the same level of communication effectiveness with their physicians using IT-mediated communication as they would in comparable FtF interactions, but patients view FtF communication to be a more favorable medium than IT-mediated communication.

## Introduction

### Effective Communication Between Patients and Physicians

Effective communication with physicians is especially important for all patients and specifically for patients with chronic diseases, as these conditions require long-term continuous care [1]. Studies have shown that effective communication facilitates improvements in patients’ physical health outcomes, self-care efficacy, and self-care management [2]. According to the Centers for Disease Control and Prevention, 60% of adults in the United States suffer from at least one chronic disease and 40% suffer from two or more. These patients account for 90% of the US $3.3 trillion annual health care costs. Managing the symptoms of chronic disease can help to reduce these costs significantly.

In recent years, patients’ and providers’ use of technology has been gaining more prevalence as patients communicate more regularly with their physicians using technology [3] and an emphasis has been placed on telemedicine. According to a report by the American College of Surgeons [4], physicians no longer make house calls, and for a growing number of patients, text messaging and telemedicine have become alternatives to phone calls and traditional office visits. Several studies have shown patients’ use of email, patient portals, and secure messaging to communicate with their physicians [5]. Research has also examined how information technology (IT)–mediated communication among patients with chronic disease can improve their health outcomes [6,7] and how media differ in effectiveness according to the communication process for which they are used (eg, scheduling an appointment and discussing acute symptoms). However, few studies have examined if patients’ perceptions of communication effectiveness differ between IT-mediated communication and face-to-face (FtF) communication. Existing research mainly examined differences between FtF communication and video and voice communication [8-10]. Research about patients’ perceptions of text-based IT-mediated communication is limited. Therefore, it is unclear if patients perceive the quality of care that is delivered FtF differently from text-based communication. As text-based IT-mediated communication is gaining more popularity [11], it is imperative to investigate how it impacts effective patient-physician communication. This gap in the literature creates an opportunity for health IT scholars to understand patients’ perspectives regarding the effectiveness of text-based IT-mediated communication. Thus, we examine if patients’ perceptions of communication effectiveness differ for FtF communication versus text-based IT-mediated communication.

Effective patient-physician communication is also an important factor in patient satisfaction, self-care efficacy, and perceived health management outcomes. Patients who experience effective communication with providers report greater satisfaction [12], self-care efficacy [13], and perceived health management outcomes [14]. These outcomes of effective communication are important, as patients who manage their health can improve their quality of life and reduce health care costs. Therefore, we also examine how perceptions of communication effectiveness facilitate patient satisfaction, self-care efficacy, and health management outcomes.

To date, researchers have studied the design, adoption, and use of information systems in health care extensively from the organizational and health provider perspective. However, there is paucity in understanding how the use of these emerging technologies can be compared with the use of traditional FtF models of care among patients. As effective communication can improve the quality of care for patients by empowering their self-care efficacy and health management, it is critical to understand patients’ perceptions of effective communication using different channels and how it impacts their satisfaction, self-care efficacy, and health management outcomes. This study begins by reviewing relevant communication theory and health communication literature to understand the role that media play in patients’ perceptions of effective communication. Next, it presents 2 competing models of effective communication, followed by the details and results of a national cross-sectional survey that tested them.

### Background

IT-mediated communication has the potential to improve cost-effectiveness, quality, and accessibility of health care services [15-18]. Several studies have reported on telemedicine and telehealth in terms of its acceptance [19,20], adoption [21-23], compliance [24], quality [25], and trust [26]. Yet, it is unclear how the quality of patient-physician interactions differs between traditional FtF interactions and text-based interactions. Most research that has examined differences in FtF interactions and IT-mediated interactions has compared video and phone consultations with FtF consultations among healthy patients. For instance, research has shown that simulated patients who either had video or FtF consultations with medical interns did not experience differences in satisfaction, perceived information exchange, interpersonal relationship building, or perceived shared decision making [9]. Similarly, patients who received an FtF, a phone, or a video consultation reported no differences in distress levels after the consultation [27]. Moreover, small samples of patients who were screened for neurocognitive problems (N=8) or took part in cognitive interventions for the elderly (N=11) using video consultations or FtF consultations experienced no differences in cognitive or diagnostic outcomes [28,29], respectively, although communication effectiveness was not assessed. The literature is promising and suggests that there are no significant differences in the quality of care that is delivered using FtF, video, or phone consultations. At the same time, it is unclear if the same results would hold when comparing text-based channels and FtF communication among patients with chronic diseases who require regular communication with their providers and use text-based communication with their providers [3].

### Cues-Filtered-In and Cues-Filtered-Out Perspectives

Frameworks that are useful for understanding the differences in communication effectiveness between FtF communication and IT-mediated communication are the cues-filtered-out and the cues-filtered-in perspectives. The cues-filtered-out perspective [30] assumes that IT-mediated communication hinders effective communication because of its reduced nonverbal cues (gestures, smiles, pats on the back, nods to show attentiveness, and eye contact), in comparison with channels that offer the transmission of more nonverbal cues such as the phone, videoconferencing, and FtF communication. A systematic review has shown the importance of nonverbal cues in patient-provider interaction in that when doctors make appropriate eye contact, do not interrupt patients, or pay attention to patients’ nonverbal signals, patients’ objective (eg, blood pressure) and subjective (eg, pain scores) health care outcomes improve [2]. The cues-filtered-out perspective echoes concerns about the use of telemedicine, such as the depersonalization of care, lack of physical presence, inhibition of patient participation, and physician dominance of the medical encounter [31].

One theory that falls in the cues-filtered-out perspective is the social presence theory [32]. This theory posits that media differ in their capacity to transmit nonverbal and verbal information; therefore, the less cues a medium can transmit, the less warmth and involvement patients and providers experience with one another. Indeed, a systematic review about patients’ experiences with remote monitoring for chronic conditions showed that patients view remote monitoring as jeopardizing interpersonal connections with their providers and do not want remote monitoring to replace FtF interactions [33]. Similarly, patients have reported missing FtF interaction with their providers when they receive telemedicine [34]. The abovementioned research suggests that patients experience less connection with their providers via telemedicine and do not want telemedicine to replace FtF communication. These results are in line with the cues-filtered-out perspective. Research has yet to show these same findings among text-based IT-mediated communication.

The cues-filtered-out perspective is in contrast to the cues-filtered-in perspective. In light of inconsistent findings for the cues-filtered-out perspective, a competing theory of social information processing was developed [35], thus creating the cues-filtered-in perspective. The cues-filtered-in perspective assumes that people adapt to the medium to achieve effective communication, regardless of the number of cues a medium transmits. This theory posits that patients and providers using IT-mediated communication can achieve the same outcomes as FtF communication, if interaction time is not restricted. Per the social information processing theory, patients and providers will use more verbal cues to achieve quality interactions and to exchange the same amount of information as they would if they were communicating FtF. In line with the social information processing theory, research has shown that simulated patients who used video, phone, or FtF communication with medical students in medical consultations reported no differences in patient satisfaction, information exchange, interpersonal relationship building, or shared decision making [9]. Similarly, patients who used either FtF communication or a video consultation with their physicians reported no differences in their physician’s ability to develop rapport, use shared decision making, and/or promote patient-centered communication [31]. The abovementioned research suggests that patients do not perceive significant differences in communication using the phone, video, or FtF medical consultations, which is in line with the cues-filtered-in perspective. At the same time, research has yet to show this empirically among text-based IT-mediated communication.

### Communication Effectiveness, Satisfaction, Self-Care Efficacy, and Health Management Outcomes

In addition to examining differences in patients’ perceptions of communication effectiveness, this study also examines differences in patient satisfaction, self-care efficacy, and health management. Effective patient-physician communication is an important factor in patient satisfaction. A large-scale intervention has shown that communication skills training for 1537 physicians improved patients’ satisfaction with provider communication [12]. At the same time, a systematic review of studies about patient satisfaction with interactive video consultations revealed mixed feelings about video consultations: Patients appreciate the accessibility of expert care, less travel, and reduced waiting times, but patients do not like communicating with their provider using video consultations [36]. Notably, this result is the opposite of the aforementioned studies that found that patients reported no differences in patient satisfaction when they used video, phone, or FtF communication in medical consultations with providers [9,31]. This research aimed to address these mixed results and advance the understanding regarding patients’ perceptions of satisfaction.

Communication effectiveness is also important for self-care efficacy and health management. Patients who experience effective communication with providers (eg, feel listened to, respected, and that their provider explains things clearly) are more motivated to take care of themselves. Similarly, patients who have quality relationships with their providers (eg, “My provider listens carefully to me” and “It is easy to communicate with my provider”) report greater levels of self-care efficacy and health care management [2,13]. Indeed, patients who have diabetes and effective provider communication report greater insulin adherence [37] and glycemic control [14]. We investigated the impact of communication effectiveness on patient satisfaction, self-care efficacy, and health management.

## Methods

### Research Model and Construct Development

To examine the differences in patients’ perceptions of FtF communication and IT-mediated communication, we apply 2 competing perspectives to advance the understanding regarding the impact of patients’ perceptions of effective communication and its impact on patient satisfaction, self-care efficacy, and health management outcomes. Per the cues-filtered-in perspective, patients should experience no differences in effective communication using IT-mediated communication (vs FtF communication) with their physicians, and, in turn, patients should report no differences in positive relationships between effective communication and self-care efficacy (H1A=H1B), effective communication and patient satisfaction (H2A=H2B), and effective communication and health management outcomes (H3A=H3B) using IT-mediated communication (vs FtF communication) with their physicians. Conversely, per the cues-filtered-out perspective, patients should experience more effective communication using FtF communication (vs IT-mediated communication) with their physicians, and, in turn, patients should report stronger positive relationships between effective communication and self-care efficacy (H1A<H1B), effective communication and patient satisfaction (H2A<H2B), and effective communication and health management outcomes (H3A<H3B) using FtF communication (vs IT-mediated communication) with their physicians.

Another important aspect of effective communication with providers is patients’ perception of media effectiveness. Media effectiveness refers to the degree to which patients perceive that a specific medium helps them accomplish their communication goal. Media effectiveness is operationalized using the same scale as communication effectiveness (eg, unsuccessful to successful, inefficient to efficient, and inappropriate to appropriate). However, we rephrased the questions to ask how effective the mode of communication is (media effectiveness), rather than how effective the interaction with one’s provider is (communication effectiveness). As such, we are using this construct to separate *communication effectiveness with providers* from *communication effectiveness with a communication mode* to aid understanding about patients’ perceptions of communication effectiveness with their providers using different media. Patients might describe interactions with their physicians as effective because they accomplish their communication goals, but they might not describe the mode of communication they used with their physician as effective because they might have preferred to use another mode of communication. Thus, we are attempting to differentiate between the different aspects of patients’ perceived communication effectiveness (PCE) by parsing media effectiveness from communication effectiveness.

A similar concept to media effectiveness is media richness or a medium’s capacity to facilitate shared understanding, as a rich medium facilitates insight and rapid understanding [38]. The media richness theory [39] operationalizes media richness based on objective characteristics of the medium, such as the speed of feedback, personal focus, the number of cues, and the ability to use natural language. As defined, media richness does not account for patients’ perceptions of media effectiveness because it refers to intrinsic characteristics of a medium. As such, we are using the media effectiveness construct to understand any differences between patients’ perceptions of communication effectiveness with providers, rather than the media richness construct.

The cues-filtered-out and cues-filtered-in perspectives offer 2 competing predictions for differences in perceived media effectiveness in physician interactions. Per the cues-filtered-in framework, patients should experience no differences in media effectiveness using IT-mediated communication (vs FtF communication) with their physicians, and, in turn, patients should report no differences in positive relationships between media effectiveness and self-care efficacy (H4A=H4B), media effectiveness and patient satisfaction (H5A=H5B), and media effectiveness and health management outcomes (H6A=H6B) using IT-mediated communication (vs FtF communication) with their physicians. Conversely, per the cues-filtered-out framework, patients should experience greater media effectiveness using FtF communication (vs IT-mediated communication) with their physicians, and, in turn, patients should report stronger positive relationships between media effectiveness and self-care efficacy (H4A<H4B), media effectiveness and patient satisfaction (H5A<H5B), and media effectiveness and health management outcomes (H6A<H6B) using FtF communication (vs IT-mediated communication) with their physicians.

**Figure 1:**
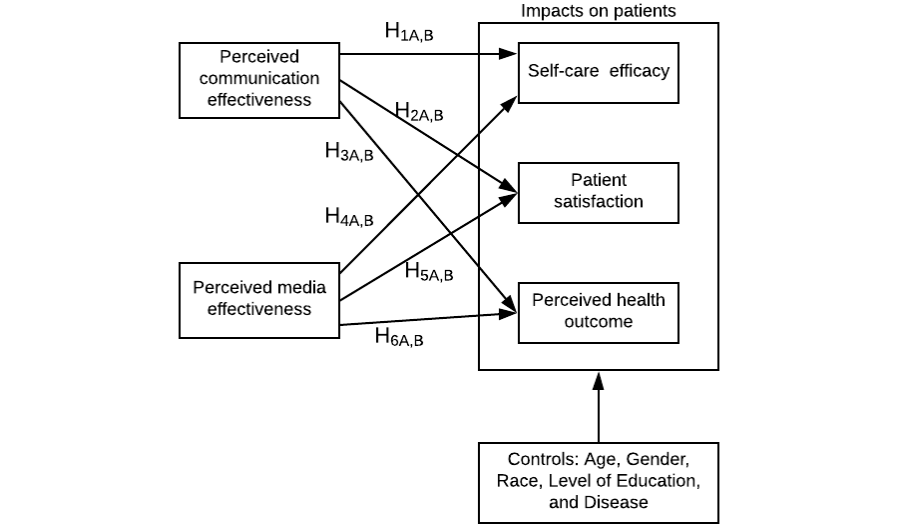
Effective communication comparison (face-to-face communication vs text-based information technology–mediated communication).

To investigate these competing predictions, we first test the proposed model (Figure 1) separately for text-based IT-mediated communication (group A) and FtF communication (group B). Then, we compare the coefficients of PCE and perceived media effectiveness between the 2 groups in the Results section.

### Data Collection

We designed and used a web-based questionnaire to survey a national sample of patients. To collect the data, we hired Qualtrics Panels. Qualtrics Panels is a market research company in the United States that partners with more than 20 sample providers to supply a network of diverse, quality respondents across the country. We used quota-based sampling to recruit approximately equal sample sizes of participants who communicated with their physician using text-based IT communication and FtF communication. Eligibility criteria included being aged at least 18 years, being located in the United States, and having engaged in communication with their physician in the previous month. Eligible participants in Qualtrics’ panel were sent an incentivized invitation with the study link in various ways, such as email, a panel portal, and text messages. Participants were first presented with an informed consent document. After agreeing to participate, they began the survey questions. Qualtrics determines the monetary incentives for taking the survey, such as cash, gift cards, and vouchers. To avoid self-selection bias, survey invitations do not include specific details about the content of the survey and are kept very general. We ensured to use neutral wordings to develop the survey and used the semantic scale to avoid any common method bias. We found that none of the correlation values among the constructs exceed 0.90 [40]. Correlation matrix is provided in Multimedia Appendix 1.

## Results

### Characteristics of Participants

In total, 349 participants completed the survey, from which we used 345 complete usable responses. Participants’ ages ranged from 35 to 85 years (mean 63, SD 9), and 45.2% (156/345) participants were male. A screening requirement of the survey was that all participants have been regularly communicating with their primary care physician over the past month. Of the 345 participants, 86 (24.9%) reported having diabetes, 69 (20.0%) reported arthritis, 52 (15.0%) reported heart disease, 34 (9.8%) reported pulmonary diseases, 28 (8.1%) reported psychiatric disorder, 17 (4.9%) reported hypertension, 17 (4.9%) reported chronic inflammatory disease, 16 (4.6%) reported thyroid, 12 (3.5%) reported immune system disorder, 7 (2.0%) reported hypertension, and 7 (2.0%) reported renal disease. For the most frequent mode of communication with physicians, of the 345, participants 134 (38.8%) reported FtF communication, whereas 211 (61.1%) participants reported the use of technology, including 152 (44.0%) patient portals, 121 (35.0%) email, and 72 (20.8%) text messaging. Of the 345 respondents, 45 (13.0%) participants completed a 2-year degree, 69 (20.0%) participants completed a 4-year degree, 72 (20.8%) participants earned a professional degree, and 145 (42.0%) participants attended some college.

### Measurement Model

We used R software (R Project for Statistical Computing) and the Lavaan package to analyze the data. To test the proposed model (Figure 1), we measured PCE using 11 bipolar semantic differential scales [41]. Example items include insufficient/sufficient, adverse/beneficial, inadequate/adequate, unsuccessful/successful, useless/useful, disadvantageous/advantageous, and inefficient/efficient. We used the same scales to measure perceived media effectiveness. For the outcome variables including patient satisfaction, self-care efficacy, and perceived health management outcomes, we adapted survey questions from previous studies and used a semantic scale ranging from 1 (strongly disagree) to 5 (strongly agree) to measure each construct. The measurement properties of each construct are reported in Table 1.

**Table 1:** Psychometric properties of measurement model.

Constructs	Description	Number of items	Cronbach alpha	Average variance extracted	Source
Perceived media effectiveness	Patients’ perception of medium’s capacity to facilitate shared understanding	10	.94	0.83	Spitzberg [41]
Perceived communication effectiveness	The degree to which patients accomplished their communication goal	10	.95	0.84	Spitzberg [41]
Patient satisfaction	The degree to which patients experienced interest, accomplishment, and overall satisfaction in conversation with their provider	5	.93	0.82	Hecht [42]
Self-care efficacy	The degree to which the patients feel confident in managing their own care	7	.87	0.70	Plotnikoff et al [43]
Perceived health outcome	Patients’ expectations of positive physical and self-evaluative outcomes	4	.91	0.72	Anderson et al [44]

We compared the proposed model for 2 groups based on the respondent’s most frequent mode of communication with their physicians: group A (IT-mediated communication) versus group B (FtF communication). Following the common criteria suggested in the literature [45,46], we examined the composite reliabilities of each construct and average variance extracted to ensure that the instrument had adequate reliability and convergent validity. Table 1 presents these statistics.

### Hypothesis Testing

To evaluate the model’s fit, we developed a structural equation model and tested for multigroup comparison while controlling for age, gender, race, the level of education, and the type of disease of the respondents. The results are presented in Table 2. To compare the 2 groups, we calculated a separate model for each group. If we assume that the residual values from the 2 models are normally distributed, we can test the hypothesis that the coefficient of PCE in group A is equal to the same coefficient in group B (ie, beta_PCE(A)_=beta_PCE(B)_) versus the alternative that they are unequal. The test statistic follows the Student *t* distribution [44] with *v* degrees of freedom where *v*=*n*_1_+*n*_2_−4 (*n*_1_: sample size of group A and *n*_2_: sample size of group B). The results of the group comparison analysis are presented in Table 3.

**Table 2:** Results for information technology–mediated communication and face-to-face communication groups.

Dependent and independent variables		Group A: information technology–mediated communication						Group B: face-to-face communication						
			Coefficient		SE		*P* value			Coefficient		SE		*P* value
**Self-care efficacy**														
	Perceived communication effectiveness (H1)		0.116		0.025		<.001			0.167		0.029		<.001
	Perceived media effectiveness (H4)		0.079		0.023		.001			0.158		0.026		<.001
	Age		0.060		0.033		.07			0.312		0.667		.64
	Gender		0.255		0.597		.67			0.036		0.038		.35
	Level of education		0.450		0.286		.12			−0.128		0.267		.63
**Patient satisfaction**														
	Perceived communication effectiveness (H2)		0.236		0.016		<.001			0.349		0.025		<.001
	Perceived media effectiveness (H5)		0.166		0.017		<.001			0.281		0.027		<.001
	Age		0.047		0.021		.03			0.062		0.033		.06
	Gender		−0.35		0.383		.36			0.588		0.577		.31
	Level of education		0.309		0.184		.09			0.595		0.231		.01
**Perceived health outcome**														
	Perceived communication effectiveness (H3)		0.126		0.017		<.001			0.171		0.025		<.001
	Perceived media effectiveness (H6)		0.078		0.016		<.001			0.139		0.024		<.001
	Age		−0.033		0.022		.14			0.041		0.033		.21
	Gender		0.275		0.403		.49			0.758		0.576		.19
	Level of education		0.236		0.193		.23			0.19		0.23		.41

**Table 3:** Model comparison across groups.

Hypotheses	*t* value (*df*)	*P* value
H1A^a^ versus H1B^b^	1.362 (333)	.17
H2A versus H2B	3.859 (333)	<.001
H3A versus H3B	1.518 (333)	.13
H4A versus H4B	2.261 (333)	.02
H5A versus H5B	3.578 (333)	<.001
H6A versus H6B	2.093 (333)	.04

^a^A: information technology–mediated group.

^b^B: face-to-face group.

The results showed that among both groups, PCE and perceived media effectiveness had a significant positive impact on patients’ self-efficacy (H1A, H1B, H4A and H4B), patient satisfaction (H2A, H2B, H5A and H5B), and perceived health management outcomes (H3A, H3B, H6A and H6B). The data also revealed that there is no significant difference in PCE between FtF communication and IT-mediated communication, yet there is a significant difference between the 2 groups in terms of perceived media effectiveness. Patients perceive FtF communication to be more effective in improving their self-efficacy than IT-mediated communication.

Additional results found that both PCE and media effectiveness have a significant impact on improving patients’ satisfaction. There is also a significant difference in the impact of PCE and media effectiveness on patient satisfaction. The impact of communication effectiveness and media effectiveness on patient satisfaction is significantly higher for FtF communication in comparison with IT-mediated communication.

Furthermore, the results revealed that PCE and media effectiveness significantly improved patients’ perceived health outcomes. Interestingly, there is no significant difference in the impact of PCE on perceived health outcomes across groups. However, there is a significant difference in the impact of perceived media effectiveness on perceived health outcomes. Patients who communicate FtF revealed a significantly higher association between media effectiveness and perceived health outcomes.

To further understand the differences in PCE and perceived media effectiveness, we compared the IT-mediated group A’s and the FtF communication group B’s responses for each scale item using *t* tests. We found no significant difference between FtF communication and IT-mediated communication groups in items that form PCE. We did, however, find significant differences between FtF communication and IT-mediated communication groups in items that formed perceived media effectiveness. There were significant differences in perceptions of media effectiveness in terms of sufficiency, adequacy, advantage, favorableness, and suitability. Patients who primarily used FtF communication (vs IT-mediated communication) assigned significantly higher scores to the abovementioned items but assigned no significant difference between the 2 groups in terms of media appropriateness, benefit, success, usefulness, and efficiency. The test results are presented in Table 4.

**Table 4:** Group comparison for perceived communication effectiveness and perceived media effectiveness.

Semantic scale	Perceived communication effectiveness		Perceived media effectiveness	
	*t* value (*df*)	*P* value	*t* value (*df*)	*P* value
Inappropriate: appropriate	−0.53 (333)	.598	−1.24 (333)	.21
Insufficient: sufficient	−0.25 (333)	.80	−2.62 (333)	.01
Adverse: beneficial	−0.97 (333)	.33	−1.70 (333)	.09
Inadequate: adequate	−1.11 (333)	.27	−3.23 (333)	.001
Unsuccessful: successful	−0.63 (333)	.53	−1.74 (333)	.08
Useless: useful	−1.07 (333)	.29	−1.76 (333)	.08
Disadvantageous: advantageous	−0.03 (333)	.98	−2.47 (333)	.01
Unfavorable: favorable	−1.18 (333)	.24	−2.43 (333)	.01
Inefficient: efficient	−0.50 (333)	.62	−1.47 (333)	.14
Unsuitable: suitable	−0.91 (333)	.37	−2.58 (333)	.01

## Discussion

### Theoretical Implications

In this study, we applied competing approaches to advance the understanding regarding patients’ perceptions of effective communication with their primary care physicians. In a national sample of patients with chronic diseases, we compared patients’ perceptions of effective communication with their provider using either text-based IT-mediated communication (email, patient portal, and messaging) or FtF communication. The primary results revealed no significant differences between patients’ perceptions of effective communication using either IT-mediated communication or FtF communication, which is in line with the cues-filtered-in perspective. Interestingly, at a more granular level, patients perceived FtF communication to be a more favorable medium than IT-mediated communication, which is in line with the cues-filtered-out perspective. As a result, differences in perceived media effectiveness impacted differences in patients’ self-care efficacy, satisfaction, and perceived health management outcomes between patients who used either FtF communication or IT-mediated communication with their physicians. The results imply that patients can achieve the same level of communication effectiveness with their physicians using IT-mediated communication as they would in comparable FtF interactions, but patients view FtF communication to be a more favorable medium than IT-mediated communication.

The results are promising as they show that as patients increasingly use different forms of IT-mediated communication, such as patient portals, email, and text messaging, to communicate with their physicians, patients will continue to adapt to the medium and achieve the same level of effectiveness, satisfaction, self-care efficacy, and health care outcomes as they would in comparable FtF interactions with their physicians. At the same time, the data show that although patients report no differences in effective communication with their physicians using IT-mediated communication or FtF communication, patients report FtF communication as a more effective mode of communication relative to IT-mediated communication. In turn, the positive relationships between media effectiveness and self-care efficacy, patient satisfaction, and perceived health management were stronger for patients who used FtF communication.

### Practical Implications

The results of this study are consistent with previous research that has shown that patients generally describe their experience with telehealth positively, yet they still value their FtF contact with health care professionals [32,33]. Patients with chronic diseases have reported many benefits of IT-mediated communication, such as improved self-management, shared decision making, better access to health care, and peace of mind. However, these potential benefits are often balanced against concerns about losing interpersonal contact. Patients often view IT-mediated communication as jeopardizing interpersonal connections with their providers and an unsuitable replacement for FtF interactions. These concerns are evident in the data, as further exploration of the underlying items that led to differences in perceived media effectiveness between FtF communication and IT-mediated communication showed that patients perceived FtF communication as a significantly more sufficient, adequate, advantageous, and favorable medium than IT-mediated communication. Notably, there were no other significant differences between FtF communication and IT-mediated communication in semantic scales that patients used to describe media effectiveness such as usefulness, success, appropriateness, and beneficial. These results suggest that patients perceive IT-mediated communication to be an effective medium for accomplishing communication goals with physicians (eg, share blood pressure data and make an appointment), but they prefer FtF communication and view it as a more suitable and favorable medium.

These mixed findings often lead researchers to infer that the sustained use of IT-mediated communication will be ensured with occasional FtF visits to physicians [47]. Nonetheless, these findings suggest a fundamental difference in how researchers and practitioners should approach patients’ adoption and use of telemedicine. More research needs to be done to shift the focus from examining the usefulness of the technology to examining how best to educate users to use the technology to improve relationships with physicians. Telemedicine holds great potential for reducing the variability of diagnoses as well as improving clinical management and delivery of health care services by enhancing access, quality, efficiency, and cost-effectiveness [48,49]. Furthermore, evidence points to important socioeconomic benefits to patients, families, health practitioners, and the health system, including enhanced patient-physician communication opportunities [50]. IT-mediated communication has yet to be consistently employed in the health care system to deliver routine services. Creating effective relationships between patients with chronic diseases and physicians is important. When patients and physicians work together to determine optimal treatment plans in a value-centered manner, this significantly improves patients’ confidence in self-care, satisfaction, and self-care management. These improvements can happen through text-based IT-mediated communication [24]. However, there is a need to educate patients about the effectiveness of IT-mediated communication. Managerial multidisciplinary efforts that draw expertise from communication sciences, health informatics and IT, public health, and health management and policy are required to ensure that telemedicine and electronic health systems are designed with a patient-centered focus and with attention to educating patients about how to use text-based technology to communicate with their physicians. Patient-physician communication will continue to evolve with time, both as a byproduct of technological advances as well as shifting societal values. In urban or developed areas, hospital beds are mainly occupied by patients with chronic diseases, decreasing bed availability for other patients in need. Recently, Centers for Medicare and Medicaid Services published a proposal for the management of patients with chronic illness that would allow physicians to be paid for non-FtF encounters. The realization of these innovative initiatives calls for changing the provider culture and workflow systems to allow the full incorporation of telemedicine into traditional care. Furthermore, patients need to be educated about how to interact with and use these technologies to communicate effectively with their providers and to manage their own care.

### Conclusions

In this study, we investigated differences in patients’ perceptions of IT-mediated communication and FtF communication. The results support that there is no significant difference in effective communication for using technology versus FtF communication. However, patients perceive FtF communication as a significantly more favorable, suitable, and sufficient medium in comparison with IT-mediated communication. More research about how to educate patients to use technology and how to use technology to improve relationships with physicians is needed.

## Appendix

Multimedia Appendix 1 Correlations matrix.

## Abbreviations

FtF: face-to-face
IT: information technology
PCE: perceived communication effectiveness

## References

[1] World Health Organization (2018). Noncommunicable Diseases Country Profiles 2018.

[2] Kelley JM, Kraft-Todd G, Schapira L, Kossowsky J, Riess H (2014). The influence of the patient-clinician relationship on healthcare outcomes: a systematic review and meta-analysis of randomized controlled trials. PLoS One.

[3] Zulman DM, Jenchura EC, Cohen DM, Lewis ET, Houston TK, Asch SM (2015). How can eHealth technology address challenges related to multimorbidity? Perspectives from patients with multiple chronic conditions. J Gen Intern Med.

[4] Shipper ES, Hardaway JC, Garvey EM, Logghe H (2016). Talking through time: trends in communication and the evolving patient-physician relationship. Bull Am Coll Surg.

[5] Lee JL, Choudhry NK, Wu AW, Matlin OS, Brennan TA, Shrank WH (2016). Patient use of email, Facebook, and physician websites to communicate with physicians: a national online survey of retail pharmacy users. J Gen Intern Med.

[6] Knox L, Rahman RJ, Beedie C (2017). Quality of life in patients receiving telemedicine enhanced chronic heart failure disease management: A meta-analysis. J Telemed Telecare.

[7] Vatnøy TK, Thygesen E, Dale B (2017). Telemedicine to support coping resources in home-living patients diagnosed with chronic obstructive pulmonary disease: patients' experiences. J Telemed Telecare.

[8] Narasimha S, Madathil KC, Agnisarman S, Rogers H, Welch B, Ashok A, Nair A, McElligott J (2017). Designing telemedicine systems for geriatric patients: a review of the usability studies. Telemed J E Health.

[9] Tates K, Antheunis ML, Kanters S, Nieboer TE, Gerritse MB (2017). The effect of screen-to-screen versus face-to-face consultation on doctor-patient communication: an experimental study with simulated patients. J Med Internet Res.

[10] Zahedi FM, Walia N, Jain H (2016). Augmented virtual doctor office: theory-based design and assessment. J Manag Inf Syst.

[11] Horwitz LI, Detsky AS (2011). Physician communication in the 21st century: to talk or to text?. J Am Med Assoc.

[12] Boissy A, Windover AK, Bokar D, Karafa M, Neuendorf K, Frankel RM, Merlino J, Rothberg MB (2016). Communication skills training for physicians improves patient satisfaction. J Gen Intern Med.

[13] Eton DT, Ridgeway JL, Linzer M, Boehm DH, Rogers EA, Yost KJ, Rutten LJ, Sauver JL, Poplau S, Anderson RT (2017). Healthcare provider relational quality is associated with better self-management and less treatment burden in people with multiple chronic conditions. Patient Prefer Adherence.

[14] Linetzky B, Jiang D, Funnell MM, Curtis BH, Polonsky WH (2017). Exploring the role of the patient-physician relationship on insulin adherence and clinical outcomes in type 2 diabetes: Insights from the MOSAIc study. J Diabetes.

[15] Chiasson MW, Davidson E (2004). Pushing the contextual envelope: developing and diffusing IS theory for health information systems research. Inform Organ.

[16] Devaraj S, Kohli R (2000). Information technology payoff in the health-care industry: a longitudinal study. J Manag Inf Syst.

[17] McCullough JS, Casey M, Moscovice I, Prasad S (2010). The effect of health information technology on quality in US hospitals. Health Aff (Millwood).

[18] Schoen C, Davis K, How SK, Schoenbaum SC (2006). US health system performance: a national scorecard. Health Aff (Millwood).

[19] Chau PY, Hu PJ (2002). Investigating healthcare professionals’ decisions to accept telemedicine technology: an empirical test of competing theories. Inform Manag.

[20] Hu PJ, Chau PY, Sheng OR, Tam KY (1999). Examining the technology acceptance model using physician acceptance of telemedicine technology. J Manag Inf Syst.

[21] Cho S, Mathiassen L, Robey D (2007). Dialectics of resilience: a multi–level analysis of a telehealth innovation. J Inf Technol.

[22] LeRouge C, Garfield M (2013). Crossing the telemedicine chasm: have the US barriers to widespread adoption of telemedicine been significantly reduced?. Int J Environ Res Public Health.

[23] Rajan B, Tezcan T, Seidmann A (2019). Service systems with heterogeneous customers: investigating the effect of telemedicine on chronic care. Manag Sci.

[24] Paré G, Jaana M, Sicotte C (2007). Systematic review of home telemonitoring for chronic diseases: the evidence base. J Am Med Inform Assoc.

[25] LeRouge C, Hevner AR, Collins RW (2007). It's more than just use: an exploration of telemedicine use quality. Decis Supp Syst.

[26] Paul DL, McDaniel Jr RR (2004). A field study of the effect of interpersonal trust on virtual collaborative relationship performance. Manag Inf Syst Q.

[27] Idan A, Wallach HS, Almagor M, Waisman Y, Linn S (2015). Mediated telemedicine vs face-to-face medicine: efficiency in distress reduction. J Multimodal User Interfaces.

[28] Grosch MC, Weiner MF, Hynan LS, Shore J, Cullum CM (2015). Video teleconference-based neurocognitive screening in geropsychiatry. Psychiatry Res.

[29] Poon P, Hui E, Dai D, Kwok T, Woo J (2005). Cognitive intervention for community-dwelling older persons with memory problems: telemedicine versus face-to-face treatment. Int J Geriatr Psychiatry.

[30] Culnan MJ, Markus ML, Jablin FM, Putnam LL, Roberts KH, Porter LW (1987). Information technologies. Handbook of Organizational Communication: An Interdisciplinary Perspective.

[31] Agha Z, Schapira RM, Laud PW, McNutt G, Roter DL (2009). Patient satisfaction with physician-patient communication during telemedicine. Telemed J E Health.

[32] Short J, Williams E, Christie B (1976). The Social Psychology of Telecommunications.

[33] Walker RC, Tong A, Howard K, Palmer SC (2019). Patient expectations and experiences of remote monitoring for chronic diseases: systematic review and thematic synthesis of qualitative studies. Int J Med Inform.

[34] Gorst SL, Coates E, Armitage CJ (2016). 'It's sort of a lifeline': chronic obstructive pulmonary disease patients' experiences of home telehealth. Health Psychol.

[35] Walther JB (1992). Interpersonal effects in computer-mediated interaction. Commun Res.

[36] Mair F, Whitten P (2000). Systematic review of studies of patient satisfaction with telemedicine. Br Med J.

[37] White RO, Eden S, Wallston KA, Kripalani S, Barto S, Shintani A, Rothman RL (2015). Health communication, self-care, and treatment satisfaction among low-income diabetes patients in a public health setting. Patient Educ Couns.

[38] Daft RL, Lengel RH, Trevino LK (1987). Message equivocality, media selection, and manager performance: Implications for information systems. Manag Inf Syst Q.

[39] Dennis AR, Kinney ST (1998). Testing media richness theory in the new media: the effects of cues, feedback, and task equivocality. Inf Syst Res.

[40] Muthen LK, Muthen BO (2015). Muthén & Muthén, Mplus.

[41] Spitzberg BH, Tardy CH (1988). Communication competence: measures of perceived effectiveness. Communication and Information Science. A Handbook for the Study of Human Communication: Methods and Instruments for Observing, Measuring, and Assessing Communication Processes.

[42] Hecht ML (1978). Measures of communication satisfaction. Human Comm Res.

[43] Plotnikoff RC, Lippke S, Johnson ST, Courneya KS (2010). Physical activity and stages of change: a longitudinal test in types 1 and 2 diabetes samples. Ann Behav Med.

[44] Anderson ES, Winett RA, Wojcik JR (2007). Self-regulation, self-efficacy, outcome expectations, and social support: social cognitive theory and nutrition behavior. Ann Behav Med.

[45] Chin WW, Esposito VV, Chin W, Henseler J, Wang H (2010). How to write up and report PLS analyses. Handbook of Partial Least Squares.

[46] Henseler J, Ringle CM, Sinkovics RR, Sinkovics R, Ghauri P (2009). The use of partial least squares path modeling in international marketing. New Challenges to International Marketing.

[47] Rajan B, Seidmann A, Dorsey ER (2013). The competitive business impact of using telemedicine for the treatment of patients with chronic conditions. J Manag Inf Syst.

[48] Dupont WD, Plummer WD (1998). Power and sample size calculations for studies involving linear regression. Control Clin Trials.

[49] Craig J, Patterson V (2005). Introduction to the practice of telemedicine. J Telemed Telecare.

[50] Jennett PA, Hall LA, Hailey D, Ohinmaa A, Anderson C, Thomas R, Young B, Lorenzetti D, Scott RE (2003). The socio-economic impact of telehealth: a systematic review. J Telemed Telecare.

