# Supplementary material for: Revisiting Effective Communication Between Patients and Physicians: Cross-Sectional Questionnaire Study Comparing Text-Based Electronic Versus Face-to-Face Communication
Source: J Med Internet Res. 2020 May 13;22(5):e16965. doi: 10.2196/16965 (PMC7254277; doi:10.2196/16965)
Supplement: Multimedia Appendix 1 [file jmir_v22i5e16965_app1.docx]

## Appendix

**Correlations matrix**

|  | **Perceived Communication Effectiveness** | **Perceived Media Effectiveness** | **Self-care Efficacy** | **Patient Satisfaction** | **Perceived Health Outcome** |
| --- | --- | --- | --- | --- | --- |
|  |  |  |  |  |  |
| **Perceived Communication Effectiveness** | 1 | 0.785^**^ | 0.409^**^ | 0.747^**^ | 0.497^**^ |
| **Perceived Media Effectiveness** | 0.785^**^ | 1 | 0.348^**^ | 0.607^**^ | 0.384^**^ |
| **Self-care Efficacy** | 0.409^**^ | .348^**^ | 1 | 0.455^**^ | 0.409^**^ |
| **Patient Satisfaction** | 0.747^**^ | 0.607^**^ | 0.455^**^ | 1 | 0.692^**^ |
| **Perceived Health Outcome** | 0.497^**^ | 0.384^**^ | 0.409^**^ | .692^**^ | 1 |
| **. Correlation is significant at the *p=.01* level (2-tailed). | | | | | |
